# The Role of Benralizumab in Eosinophilic Immune Dysfunctions: A Case Report-Based Literature Review

**DOI:** 10.1155/2023/8832242

**Published:** 2023-04-24

**Authors:** Margarida Gomes, Ana Mendes, Filipa Ferreira, Joana Branco, Fernanda S. Tonin, M. Elisa Pedro

**Affiliations:** ^1^Serviço de Imunoalergologia, Hospital de Santa Maria, Centro Hospitalar e Universitário Lisboa Norte, Lisbon, Portugal; ^2^Serviço de Gastroenterologia, Hospital Professor Doutor Fernando Fonseca, EPE, Amadora, Portugal; ^3^H & TRC-Health & Technology Research Center, ESTeSL-Escola Superior de Tecnologia da Saúde, Instituto Politécnico de Lisboa, Lisbon, Portugal

## Abstract

In the past years, the knowledge of eosinophils playing a primary pathophysiologic role in several associated conditions has led to the development of biologics targeting therapies aiming at normalizing the immune response, reducing chronic inflammation, and preventing tissue damage. To better illustrate the potential relationship between different eosinophilic immune dysfunctions and the effects of biological therapies in this scenario, here, we present a case of a 63-year-old male first referred to our department in 2018 with a diagnosis of asthma, polyposis, and rhinosinusitis and presenting a suspicion of nonsteroidal anti-inflammatory drugs' allergy. He also had a past medical history of eosinophilic gastroenteritis/duodenitis (eosinophilia counts >50 cells/high-power field HPF). The use of multiple courses of corticosteroid therapy failed to completely control these conditions. In October 2019, after starting benralizumab (an antibody directed against the alpha chain of the IL-5 cytokine receptor) as add-on treatment for severe eosinophilic asthma, important clinical improvements were reported both on the respiratory (no asthma exacerbations) and gastrointestinal systems (eosinophilia count 0 cells/HPF). Patients' quality of life also increased. Since June 2020, systemic corticosteroid therapy was reduced without worsening of gastrointestinal symptoms or eosinophilic inflammation. This case warns of the importance of early recognition and appropriate individualized treatment of eosinophilic immune dysfunctions and suggests the conduction of further larger studies on the use of benralizumab in gastrointestinal syndromes aiming at better understanding its relying mechanisms of action in the intestinal mucosa.

## 1. Introduction

Eosinophils are innate immune multifunctional leukocytes that participate in diverse inflammatory pathways, modulate innate and adaptive immune responses and act as effector cells producing tissue-destructive cationic proteins, cytokines, chemokines, and lipid mediators. These cells are associated with host defense against parasitic viral, fungal, and bacterial infections. However, recent studies demonstrated that eosinophils also play a primary pathophysiologic role in the so-called eosinophil-associated disorders (EADs), a wide variety of conditions including rhinitis, eosinophilic asthma, chronic rhinosinusitis, dermatitis, hypereosinophilic syndrome, and eosinophilic esophagitis [1, 2].

These disorders have been historically treated with corticosteroids and immunosuppressants, which are often effective at reducing eosinophil counts and associated eosinophilic inflammation but are also associated with serious long-term adverse events [3]. After 2005-2006, advances in the development of new treatments led to the approval of biological therapies, whose overall mechanism of action targets selective eosinophilopoietic cytokines or other cells interacting with or activating inflammatory pathways. Benralizumab, an antibody directed against the alpha chain of the IL-5 cytokine receptor (IL-5RA), was approved as add-on treatment for moderate to severe eosinophilic asthma grounded on the results of multicenter randomized clinical trials showing significant asthma control improvement and exacerbations reductions (moderate-high certainty of evidence) [4–6]. More recently, studies have suggested a potential additional effect of benralizumab for depleting both blood and gastrointestinal tissue eosinophilia in some patients with eosinophilic gastrointestinal disorders (EGID), which may lead to a novel clinical indication for the use of this therapy. Nonetheless, the clinical response among patients using biologics seems heterogeneous, and the possible causative link between this EAD and immunotherapy is not completely known, which requires further investigation [7–10]. These are especially important given the unmet need in this field for targeted therapies that can normalize the immune response to triggers, reduce chronic inflammation, and limit or prevent remodeling and tissue fibrosis. Currently, conventional treatment of EGID includes diet, proton pump inhibitors, and corticosteroids [11].

In this context, we aimed to briefly review the literature on the multiple aspects of eosinophilic immune dysfunctions, focus on the potential association between asthma and EGID, and share the outcomes of a case of a male patient with gastrointestinal eosinophilic syndrome, for whom benralizumab has been indicated for managing severe asthma.

## 2. Literature Review

### 2.1. Eosinophilic Immune Dysfunctions

Eosinophils, although representing only the second granulocyte subpopulation in the circulating blood (<6% of the bone marrow resident nucleated cells), are receiving a growing interest from the scientific community given their complex pathophysiological role in immune homeostasis both as effector immune cells committed to the host defense and as modulators of innate and adaptive immune responses. The most relevant mechanisms of actions are the axis between eosinophils and T-cells, more specifically T helper type 2 (Th2) cells [12]. Th2 cells can stimulate eosinophils either directly through the release of interleukin-5 (IL5), or indirectly by promoting a humoral adaptive response with a particular production of IgE, leading to a cascade of events (e.g., a network of intracellular signaling pathways) in the main tissues in which eosinophils are recruited (i.e., digestive tract, adipose tissue, lung, mammary gland, thymus, and uterus) [13].

Classically, eosinophils are associated with the combat of helminthic infections and induction of tissue damage in the context of allergies. However, a broad range of other local and systemic inflammatory diseases as well as cancer and thrombosis are being related to deranged eosinophil functions and unbalanced Th2-responses often associated with eosinophilia or eosinophil-mediated tissue damage which highlights the role of eosinophils as biomarkers. Eosinophilia refers to an absolute count of eosinophils exceeding 450–500 cells/𝜇l, while blood type eosinophilia is usually employed to define thresholds of 1500 cells/*μ*l. In this context, eosinophilic immune dysfunctions or eosinophil-associated diseases/disorders (EADs) are being currently defined as a range of heterogeneous conditions in which eosinophils are believed to play a critical pathological role in self-perpetuating inflammatory cycle of diseases [1, 2]. EADs include common illnesses such as eosinophil-related respiratory diseases (chronic rhinosinusitis, nasal polyposis, and asthma) and skin diseases (dermatitis, cellulitis, urticaria, and severe drug reactions), and other rare conditions such as hypereosinophilic syndromes (HES, defined as association of blood hypereosinophilia with established eosinophil-related organ damage), hypereosinophilia of undetermined significance (HEUS, defined as clinically silent cases of hypereosinophilia), eosinophilic granulomatosis with polyangiitis (EGPA), and eosinophilic gastrointestinal disorders (EGID). Eosinophilia can also be observed in other immune-mediated diseases such as pemphigoid, systemic sclerosis, sarcoidosis, and IgG4-related diseases [4, 14].

EADs are lifelong conditions requiring monitoring and associated with substantial burdens both for the patient and its family/caregivers (including chronic and debilitating symptoms, decreased health-related quality of life, and increased out-of-pocket costs for treatment) and for the healthcare system (need for multiple different healthcare professionals and increased emergency departments visits and hospital stays). In New Zealand, for instance, severe eosinophilic asthma patients present over 1.5 times as many respiratory treatment prescriptions and 3.6 higher asthma-related healthcare costs compared to other patients [15]. In the United Kingdom, although the prevalence of severe, uncontrolled eosinophilic asthma is low (1%), the associated health-related costs are four times greater (cost ratio 3.9 (95% IC 3.7–4.1)) [16]. Moreover, healthcare resource utilization may be particularly high in EGID, with contributing factors including diagnostic delays (poor disease recognition), repeated endoscopy, and lack of approved medications [11, 17, 18]. The median total annual cost of a single eosinophilic esophagitis case in the United States was estimated at USD $3,300 (outpatient visits, pharmacy, and endoscopy); the total health utilization costs per year vary between $350 and 950 million [19–22].

### 2.2. Linking EGIDs and Allergic Diseases

EGIDs are an umbrella term referring to primary eosinophilic disorders of the gastrointestinal tract encompassing a spectrum of diseases characterized by prominent eosinophilic inflammation affecting different regions of the gut (i.e., esophagus, stomach, small bowel, and colon or a combination) that occur in the absence of secondary causes (e.g., infections and drug reactions) [23, 24]. The strong association of EGID with allergic disorders such as asthma, dermatitis, rhinitis, and food or drug allergies has been recently recognized, and although their shared pathophysiological basis remains partly elusive (e.g., both are characterized by immunologically Th2 responses and allergic sensitizations), this feature may impact on accurate diagnostic and treatment of these conditions [6, 24]. Moreover, very few mechanistic studies specifically examine both disorders when found concomitantly in patients [25].

Most of the knowledge about the coexistence or relationship between EGIDs and asthma comes essentially from the eosinophilic esophagitis literature, probably because this is the most frequent form of EGIDs and because gastritis, enteritis, and gastroenteritis are usually considered as a whole nosologic entity given their clinical similarities. The prevalence of asthma in patients with esophagitis is estimated between 25% and 60%, while in eosinophilic gastritis or gastroenteritis cases it may reach 40%. Primary eosinophilic colitis is the least frequent disorder among EGID; asthma is reported in around 15% of these patients [25, 26]. Recent meta-analyses (21 studies, *n* = 53,592 adult and pediatric patients) confirm that individuals with EGID have significantly increased probability of having asthma (OR 3.06 (95% IC 2.01–4.66)) when compared to controls [27–30].

In this scenario, since asthma and EGID may share similar molecular pathophysiology, the possibility of using target therapies has emerged as a potential research area [25].

### 2.3. Treatment Options

EADs are typically managed with topical or systemic corticosteroids and immunosuppressants, which are initially effective in controlling eosinophilia and symptoms in many patients. However, chronic use of these therapies often leads to serious short-and long-term adverse effects and resistance, requiring second-line agents [4, 5]. For EGID, conventional treatments additionally include elimination diet, proton pump inhibitors, and esophageal dilation in patients who have developed strictures. Nonetheless, these treatments also demonstrate variable response rates and may not always provide long-term disease control [11].

In the past decade, the knowledge of eosinophil biology has led to the development of several biologics targeting eosinophils. These therapies provide the advantage of targeting specific cells or pathways, theoretically increasing efficacy, and limiting complications related to nonspecific effects of more traditional therapeutic approaches. Early research and development efforts of these biologics focused on blocking the action of IL-5; yet, the number of agents approved and under development for managing EAD- especially asthma, has significantly increased in the last years [3, 5]. A review of the mechanism of action, efficacy, and safety of different biologicals to treat asthma (i.e., benralizumab, mepolizumab, dupilumab, and reslizumab) grounded the development of clinical recommendations and practical guidelines in the field with moderate to high certainty. All these biologicals are able to reduce severe asthma exacerbations, improve asthma control, health-related quality of life, and FEV1, without reaching minimal important differences. They can also reduce the daily doses of oral corticosteroids [31, 32].

Benralizumab is a humanized recombinant monoclonal antibody of the isotype IgG1k immunoglobulin that specifically binds to the alpha chain of the IL-5 receptor expressed on eosinophils and basophils. This drug is approved since 2017 in the United States (Food and Drug Administration agency) and 2018 in Europe (European Medicines Agency) as a maintenance treatment for patients with severe asthma and an eosinophilic phenotype, grounded mostly on the clinical results from phase III pivotal multicenter randomized trials (SIROCCO, CALIMA, and ZONDA), which are also highlighted in several systematic review with meta-analyses [32–35]. The usual dose is 30 mg SC every 4 weeks for 3 doses and then every 8 weeks. Other recent studies have also demonstrated potential benefits with the use of benralizumab for managing further respiratory-related EAD such as chronic rhinosinusitis with nasal polyps [36] and chronic obstructive pulmonary disease (at higher doses) [37].  Table 1 depicts most of the ongoing clinical trials with benralizumab targeting several EADs other than asthma.

Conversely, so far only the biologic dupilumab has been approved (May 2022) by the US Food and Drug Administration (FDA) agency to treat eosinophilic esophagitis (adults and pediatric patients 12 years and older), grounded on the positive results of a phase 3 randomized, double-blind, multicenter, and placebo-controlled trial [38, 39]. Other approvals of biological treatments for these EGID indications are scarce [40]. Some recently published case reports [9, 10] demonstrated potential histological remission of gastrointestinal eosinophilia in patients under benralizumab for asthma. However, the clinical response over time seems heterogeneous and may rely on different factors (e.g., gastrointestinal segment affected), requiring further investigation and discussion on the matter.

## 3. Case Presentation

**Figure 1:**
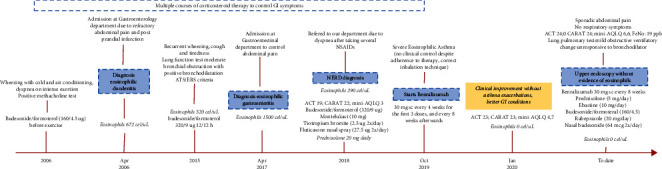
Timeline of the case report: patient journey and main outcomes.

This study followed the standards for scientific research of the Declaration of Helsinki. Informed consent was obtained from the patient, and anonymity was guaranteed (the study followed the CARE checklist 2013-Supplementary material (Available here)). The overall timeline and major events are depicted in Figure 1.

A 63-year-old male, with a diagnosis of asthma, polyposis, and rhinosinusitis was referred to our facility (Lisbon, Portugal) in 2018 due to suspicion of NSAIDs (nonsteroidal anti-inflammatory drugs) allergy and difficult to control asthma. The patient also had a past medical history of eosinophilic gastroenteritis confirmed by biopsy performed in 2006 (eosinophilic duodenitis) and later on in 2017 (gastric, duodenal, and colonic eosinophilic infiltrates).

According to the patient medical record, gastrointestinal symptoms began in 2000, when he started having sporadic abdominal pains that were relieved with proton pump inhibitors. At this time, he also had eosinophilia (1526 cell/*μ*L), but due to clinical resolution on further investigation was performed. In 2006, the patient was admitted to the Gastroenterology Department due to refractory abdominal pain and postprandial infarction. He still had blood eosinophilia (672 cell/*μ*L). Celiac disease, inflammatory bowel disease, and infection were excluded; an upper endoscopy revealed the presence of eosinophilic duodenitis. The patient underwent a course of corticosteroid therapy and was discharged with symptom resolution. In 2009, ulcerative proctitis was assumed, and he was medicated with mesalamine with resolution of symptoms.

In the same year, the patient started exercise dyspnea-later confirmed in a metacholine bronchial provocation test. At that time, budesonide/formoterol 160/4.5 *μ*g daily was indicated (regimen maintained until 2018). Between 2015 and 2018, he complained about worsening asthma symptoms, specifically cough, dyspnea, and wheezing (with cold and with little physical effort). Currently eosinophil count: 520/*μ*L.

**Figure 2:**
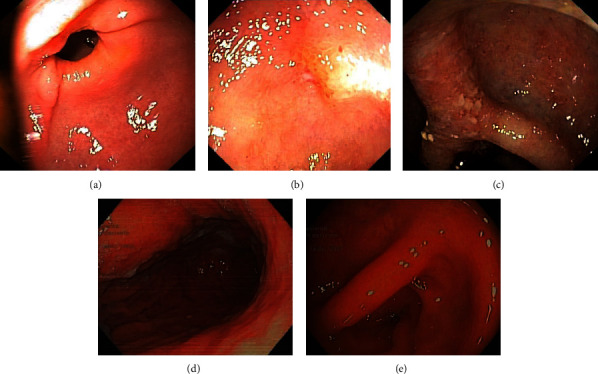
Comparison of endoscopic alterations in 2017 (a–c) and endoscopic healing in 2022 (d, e). (a) Hyperemia in the gastric antrum. (b) Erosion and edema in the terminal ileum. (c) Erosions, hyperemia, and edema in the transverse colon. (d) Normal gastric body. (e) Normal colonic mucosa in the transverse colon.

In 2017, due to uncontrolled abdominal pain, the patient was again admitted to the Gastroenterology Department showing moderate blood eosinophilia (1500 cell/*μ*L). The upper endoscopy and ileocolonoscopy, both revealed (off) esophageal, gastric, duodenal, ileal, and colonic eosinophilic inflammation with eosinophilic infiltrates (namely, >50/cell/high-power field (HPF) in the ileon and colon) besides endoscopic evidence of erosions and hyperemia in the ileocolonosocpy (Figures 2(a)–2(c)). Eosinophilic granulomatosis with polyangiitis was excluded (negative p-ANCA and C-ANCA; normal ECG, echocardiogram, and normal thoracic CT-scan. Skin biopsy also showed no vasculitis); a diagnosis of eosinophilic gastroenteritis was reached.

In 2018, the patient was once referred to our department due to suspicious NERD (NSAID exacerbated respiratory disease) as he presented dyspnea while taking nimesulide, acetylsalicylic acid, diclofenac, and metamizole. As the patient was on systemic corticosteroid therapy, it was not possible to do NSAIDs provocation test; yet, clinical history was consistent, and the diagnosis of NERD was assumed. At this time, the blood eosinophil count was 210 cells/mcL (610 in April 2017), ACT (asthma control test) 19 points, CARAT 22 points, and mini-AQLQ (mini asthma quality of life test) 3.0 points. Inhaler technique and adhesion were reviewed, and the patient was prescribed budesonide/formoterol 320/9 *μ*g twice daily, tiotropium bromide 2,5 *μ*g twice in the morning, and montelukast 10 mg daily. The results from pulmonary tests (2017) were as follows: FEV1 3.35 L, 102%; after bronchodilatation 3.76 L, 115% > 12%; FEV1/FVC 62.37%; FVC 5.35 L; after bronchodilatation 5.24 L, <2%; TLC 8.26 L, 123%; ITGG 4,36 L (normal), raw 0.31 kPA*∗*s/L (slightly increased, theoretical value for this patient 0.30 kPA*∗*s/L); normal diffusing capacity.

In 2019, it was assumed that the patient had severe eosinophilic asthma as the disease was not controlled despite adequate pharmacological therapy according to the GINA guidelines, therapeutic adherence, and correct inhalation technique. Simultaneously, the patient had worsening gastrointestinal symptoms with the need of using systemic steroids (prednisolone 20 to 40 mg daily). This scenario led to the introduction of benralizumab (October 2019) at conventional dosage (first three doses every 4 weeks and then every 8 weeks). Sixteen weeks after the first benralizumab injection, clinical improvements in both asthma and gastrointestinal symptoms without asthma exacerbation were noted. No adverse reactions were reported. Improvement in asthma scores (ACT 23 points; CARAT 23 points; mini-AQLQ 4.7 points) and a reduction in blood eosinophil count (to 0 cells/*μ*L) were evident. Additionally, since June 2020, it was possible to reduce systemic corticosteroids to 5 mg per day without worsening of gastrointestinal symptoms. In November 2021, no self-reports of both asthma and gastrointestinal symptoms were recorded (blood eosinophil count 0 cells/*μ*L).

Recently, in March 2022, the patient performed a routine upper endoscopy and ileocolonoscopy that still shows no eosinophilic inflammation (Figures 2(d) and 2(e)). He is currently prescribed with benralizumab 30 mg each 8 weeks, montelukast 10 mg, rabeprazole 20 mg, budesonide/formoterol 160/2.5 *μ*g daily, topical nasal budesonide 100 *μ*g daily, and prednisolone 5 mg. No complains of respiratory nor gastrointestinal symptoms were recorded; quality of life has improved (ACT 24 points; CARAT 24 points; mini-AQLQ 6.6). The measuring of exhalated nitric oxide did not show eosinophilic inflammation (19 ppb). Since benralizumab initiation, no hospital admissions were reported. The results from pulmonary tests, under maintenance therapy (2022), were as follows: FEV1 3.56 L, 114%; after bronchodilatation 3.95 L > 11%; FEV/FVC 64.12%; FVC 5.44 L, 137% -after bronchodilatation 5.72 L > 5%; TLC 7.76 L; 115% (see Table 2 for changing in clinical parameters).

## 4. Discussion

This case and updated literature review warn of the diverse organ manifestations of Th2 inflammation and the potential associations between asthma and EGID, whose pathophysiological mechanisms are not yet completely elucidated. Our findings additionally suggest that target therapies such as benralizumab may have an important role in treating different eosinophilic conditions.

Recognition and appropriate treatment of EGID complications (i.e., symptoms, disease activity, continuous monitoring, and maintenance treatment) by means of a multidisciplinary approach are currently of utmost importance to improve clinical outcomes and patients' quality of life [24]. Similarly, international asthma guidelines also recommend regular assessment and target treatment of comorbidity conditions such as allergic rhinitis, gastroesophageal reflux disease, and sinusitis [24]. Eosinophilic esophagitis, for instance, has been often associated with allergic diseases such as asthma, allergic rhinitis, and atopic dermatitis. It is plausible that similar mechanisms exist for EGID to affect asthma given the pulmonary-esophageal relationship in airway disease [25].

Although ideal biomarkers are still currently needed to better characterize asthma phenotypes, it is also important to recognize blood eosinophil count and its proportion with blood neutrophils and lymphocytes as the most reliable measures to predict airway eosinophilia in severe asthma and, therefore, the response to traditional treatment with inhaled corticosteroids and biologic drugs. In this context, the development, validation, and implementation of further point-of-care devices for the management of these conditions in clinical practice are recommended [41].

Moreover, translational research has identified potential pathways to target in the treatment of EGID, including Th2 inflammation-targeted systemic pathways, mast cells, and eosinophils [40]. Preliminary studies reported that cendakimab (IL-13) affect eosinophil count and patients' symptoms. The IL-5-targeting agents mepolizumab and reslizumab, commonly prescribed for severe eosinophilic asthma, showed histologic improvements in eosinophilic esophagitis (e.g., reduction in eosinophils), but failed to induce symptomatic improvement [42, 43]. The results of a phase 2 trial using lirentelimab (a monoclonal antibody targeting the eosinophil and mast cell transmembrane protein Siglec8) demonstrated a slightly dysphagia improvement in a subset of patients with eosinophilic gastritis or duodenitis [44]. Yet, these studies were only powered to assess changes in eosinophil counts rather than any clinical outcomes. Only recently (2022) dupilumab, a fully human monoclonal antibody that blocks IL-4 and IL-13 signaling, was approved by the FDA as first target treatment for eosinophilic esophagitis given the improved histologic remission (rates of around 60% when administered weekly vs. 5% in placebo; *p* < 0.001) and alleviated symptoms of the disease [38, 39]. In this context as novel therapies and clinical indications or drug repurposing begin to emerge (i.e., see Table 1 for ongoing clinical trials on the use of benralizumab), there is a growing need for the full characterization and standardization of outcomes to better understand treatment responses and to facilitate the optimization and personalization of treatment [11].

Nonetheless, different patients may present different responses to treatment. A recent study reported that benralizumab led to histological remission of eosinophilic esophagitis in a 56-year-old patient with severe asthma. Conversely, complete clinical remission was not observed, which exemplified the complex nature of EGID, its associated psychosomatic burden, and the chronification of the disease [9]. A case series of *n* = 7 patients with eosinophilic GI disorders (*n* = 7 of them (71.4%) carrying a diagnosis of asthma) also demonstrated that benralizumab completely depleted blood and GI tissue eosinophilia. However, the clinical response, while encouraging, was heterogeneous. Potential explanations for this variability include a lag between eosinophil depletion and normalization of the mucosa and the involvement of other cells, such as epithelial cells or mast cells, in the pathogenesis of symptoms. Residual symptoms in some patients may reflect persistent epithelial changes in the upper GI tract [10]. In our case, the use of benralizumab also led to a significant and objective reduction of eosinophilia in peripheral blood samples as well as in the histological examination, with zero eosinophilic granulocytes (0/high power field). More importantly, we noted a reduction in patients' GI complaints/symptoms and hospital admission after treatment initiation, which may be associated with better clinical outcomes and increased quality of life for the patient and his family/caregivers and a reduced burden for the health system.

It is also important to highlight that no significant adverse events or drug discontinuation were related to benralizumab in this case report. In fact, a recent meta-analysis demonstrated that this drug has a low risk of overall adverse events (most commonly reported: headache and pyrexia), serious adverse events, asthma exacerbation, bronchitis, and sinusitis, being relatively safe in both short-and long-term treatments [35]. Moreover, recent real-world studies on the management of eosinophilic lung diseases confirmed that add-on biological therapy with benralizumab leads to market improvement of severe eosinophilic asthma (including asthma exacerbation rates, prednisone intake, asthma control test, fractional exhaled nitric oxide, quality of life, and eosinophils count) both in patients with and without comorbid chronic rhinosinusitis with nasal polyps and at no significant safety issues [45, 46].

Yet, one should be aware that the current approved dose for benralizumab (i.e., as add-on maintenance treatment in adult patients with severe eosinophilic asthma inadequately controlled despite high-dose inhaled corticosteroids plus long-acting*β*-agonists) is 30 mg by subcutaneous injection every 4 weeks for the first 3 doses and then every 8 weeks thereafter. An adjusted dose targeting EGID treatment should be further investigated as it may influence pharmacokinetics/dynamics and outcome achievement. Although a potential synergism between corticosteroids and benralizumab may exist, a recent study indicates that benralizumab causes rapid and near complete depletion of blood eosinophils in the first 24 hours after treatment with a speed of onset effect very similar to that seen with oral prednisolone (time for blood eosinophil level to decrease 50% from baseline was of 1.7 hours (0.7) for benralizumab vs. 2.5 hours (0.3) for prednisolone; *p*=0.874). Authors suggest that benralizumab may be an alternative noncorticosteroid treatment for acute exacerbations of eosinophilic asthma with the potential benefit of inhibiting eosinophilic airway inflammation for at least 30 days after one injection [47]. In our case, it was possible to reduce patients' systemic corticosteroids to 5 mg per day, without worsening of GI symptoms and within six months after benralizumab initiation.

Our findings contribute to the emerging body of data suggesting the need for further studies with a larger number of patients with past clinical history of GI syndrome on benralizumab therapy aiming at better understanding its relying mechanisms of action in the intestinal mucosa and the potential benefits for immune disorders. Given the complexity and potential heterogeneity of EGID (e.g., discordance between symptoms, histology, and endoscopic features), there is a need for comprehensive treat-to-target goals, individualized therapy, and continuous monitoring of treatment response.

This study has some limitations. Although a systematic literature review would be methodologically more robust to cover the topic of asthma and EGID conditions, we demonstrated through a narrative review that the literature is still scarce. Our discussion is grounded on the results of a case reported in our center; however, other cases may have slightly different outcomes and thus should be interpreted within their clinical contexts.

## Figures and Tables

**Table 1:** Main ongoing clinical trials with benralizumab.

Trial	Phase	Conditions	Treatments	Status
NCT03563066	II	Atopic dermatitis	Benralizumab placebo	Completed
NCT04605094	II	Atopic dermatitis	Benralizumab placebo	Active
NCT04612725	IIb	Chronic spontaneous urticaria	Benralizumab placebo	Active
NCT03183024	IV	Chronic idiopathic urticaria	Benralizumab placebo	Completed
NCT01227278	II	Chronic obstructive pulmonary disease	Benralizumab placebo	Completed
NCT02138916	III	Chronic obstructive pulmonary disease	Benralizumab placebo	Completed
NCT02155660	III	Chronic obstructive pulmonary disease	Benralizumab placebo	Completed
NCT04098718	II	Chronic obstructive pulmonary disease	Benralizumab placebo	Recruiting
NCT04053634	III	Chronic obstructive pulmonary disease	Benralizumab placebo	Recruiting
NCT05273359	II	Chronic obstructive pulmonary disease	Benralizumab placebo	Not yet recruiting
NCT03401229	III	Nasal polyps	Benralizumab placebo	Completed
NCT04185012	III	Nasal polyps	Benralizumab placebo	Recruiting
NCT03450083	II	Chronic rhinosinusitis with nasal polyps	Benralizumab placebo	Completed
NCT04157335	III	Chronic rhinosinusitis with nasal polyps	Benralizumab placebo	Recruiting
NCT02772419	II	Eosinophilic chronic rhinosinusitis	Benralizumab placebo	Completed
NCT03473977	II/III	EGID^*∗*^	Benralizumab placebo	Completed
NCT04543409	III	EGID^*∗*^	Benralizumab placebo	Active
NCT05251909	III	EGID∗	Benralizumab placebo	Recruiting
NCT04191304	III	Hypereosinophilic syndrome	Benralizumab placebo	Recruiting
NCT02130882	II/III	Hypereosinophilic syndrome	Benralizumab placebo	Not yet recruiting
NCT04157348	III	Eosinophilic granulomatous vasculitis	Benralizumab mepolizumab	Recruiting
NCT04612790	III	Bullous pemphigoid	Benralizumab placebo	Recruiting
NCT04108962	IV	Allergic bronchopulmonary aspergillosis	Benralizumab placebo	Withdrawn^*∗∗*^

^*∗*^Eosinophilic gastroenteritis or eosinophilic gastritis. ^*∗∗*^Difficult to find eligible patients.

**Table 2:** Changes in clinical parameters during the patient follow-up (2019–2022).

Main parameters	Before benralizumab (2019)	After benralizumab (to date)
Eosinophils (cell/*μ*L)	290	0
Prednisolone (mg)	20	5
ACT (points)	19	23
CARAT (points)	22	23
Mini-AQLQ (points)	3	4.7

## Data Availability

All information that grounded this case report is available within the main text, tables, and figures. Further data are available from the corresponding author upon request.

## Supplementary Materials

Supplementary Materials Completed CARE checklist for case reports.
